# Responses of the Ocular Anterior Segment and Refraction to 0.5% Tropicamide in Chinese School-Aged Children of Myopia, Emmetropia, and Hyperopia

**DOI:** 10.1155/2015/612728

**Published:** 2015-09-20

**Authors:** Ying Yuan, Zhengwei Zhang, Jianfeng Zhu, Xiangui He, Ergang Du, Kelimu Jiang, Wenjing Zheng, Bilian Ke

**Affiliations:** ^1^Shanghai Eye Disease Prevention & Treatment Center, Shanghai 200040, China; ^2^Department of Ophthalmology, Shanghai General Hospital, Shanghai Jiao Tong University School of Medicine, Shanghai 200080, China; ^3^Chinese Medicine Hospital of Zhejiang Province, Hangzhou, Zhejiang 310006, China

## Abstract

*Purpose*. To investigate the changes of anterior segment after cycloplegia and estimate the association of such changes with the changes of refraction in Chinese school-aged children of myopia, emmetropia, and hyperopia. *Methods*. 309 children were recruited and eligible subjects were assigned to three groups: hyperopia, emmetropia, or myopia. Cycloplegia was achieved with five cycles of 0.5% tropicamide. The Pentacam system was used to measure the parameters of interest before and after cycloplegia. *Results*. In the myopic group, the lenses were thinner and the lens position was significantly more posterior than that of the emmetropic and hyperopic groups in the cycloplegic status. The correlations between refraction and lens thickness (age adjusted; *r* = 0.26, *P* < 0.01), and lens position (age adjusted; *r* = −0.31, *P* < 0.01) were found. After cycloplegia, ACD and ACV significantly increased, while ACA significantly decreased. Changes in refraction, ACD, ACV, and ACA were significantly different among the three groups (*P* < 0.05, all). Changes of refraction were correlated with changes of ACD (*r* = 0.41, *P* < 0.01). *Conclusions*. Myopia presented thinner lenses and smaller changes of anterior segment and refraction after cycloplegia when compared to emmetropia and hyperopia. Changes of anterior chamber depth were correlated with refraction changes. This may contribute to a better understanding of the relationship between anterior segment and myopia.

## 1. Introduction

Myopia is a significant public health problem worldwide and is especially prominent in East Asian countries such as China and Singapore [1–6]. The incidence of myopia tends to increase in adolescence and is the main cause of low vision among the population [7]. The exact mechanism underlying development of myopia has not been well elucidated, but it is clear that the ocular refraction components including corneal, anterior segment, lens thickness (LT), vitreous chamber depth, and axial length are mismatched in myopia. The crystalline lens accounts for almost 20% of the eye's refracting power. During emmetropization of human eyes, the crystalline lens undergoes thinning and flattening [8, 9]. However, the role of the lens in myopia onset and progression is still controversial. A longitudinal study from Singapore showed that changes in LT were biphasic in different refractive status [10]. Other studies with myopia models indicated that the role of the lens in refractive error development was nonexistent [11]. Moreover, the importance of lens position (LP), defined as anterior chamber depth (ACD) + LT/2, has rarely been investigated in myopes. Therefore, we conducted this cross-sectional study to investigate the association between LT, LP, and myopia.

Evaluation of the ocular anterior segment was essential in clinical ophthalmology. During accommodation, the LT and anterior lens curvature changed which would lead to a change in anterior segment structure. The role of anterior segment in myopia development has also come to the attention of researchers currently. Previous work reported that the anterior chamber angle (ACA) and ACD increased with myopia progression [12, 13]. Meanwhile, myopes usually showed insufficient accommodation or even accommodative lag [14, 15]. However, the effect of accommodation on the anterior segment of myopia remained unclear. So we investigated the relationship between change of anterior segment and change of refraction before and after cycloplegia here.

In this study, 0.5% tropicamide was used to relax accommodation and Pentacam was used to investigate the front corneal radius, back corneal radius, ACD, ACV, and ACA in myopia, emmetropia, and hyperopia before and after cycloplegia. Subjective refraction was also measured before and after application of tropicamide. Additionally, LT and LP after cycloplegia were measured to investigate the role of lens in myopia development. The results would shed more light on the relationship between anterior segment and myopia.

## 2. Materials and Methods

### 2.1. Subjects

Three hundred and nine children from one primary school in Chongming, Shanghai, China, were invited to participate in our study. Children with eye diseases and a history of ocular trauma and surgery or who could not cooperate during Pentacam measurements were excluded. The parameters of interest were measured bilaterally but only data from the right eyes were compared. This study was approved by the Human Research Committee of Shanghai General Hospital, Shanghai Jiao Tong University School of Medicine. We obtained informed written consent from at least one legal guardian, as well as verbal consent, from each child. The study's protocol conformed to the restrictions established by the Declaration of Helsinki.

### 2.2. Ocular Examination

All participants underwent a complete ophthalmic examination which included visual acuity, slit-lamp biomicroscopy, and the refraction of noncycloplegic and cycloplegic state. Best-corrected visual acuity was assessed monocularly with linear logMAR charts if the child had an uncorrected visual acuity higher than 0.0logMAR (i.e., vision > 0.0logMAR). A Haag-Streit slit-lamp (Koeniz, Switzerland) was used to examine the anterior segment. Five cycles of tropicamide (0.5%, one drop per cycle) were administered 5 minutes apart. About 30 minutes after application of the eye drops, children without pupillary light reflex were examined by an autorefractometer (RK-F1, Canon), which generated 5 valid readings of refraction, and the median value was used for analyses.

### 2.3. Pentacam Measurement

Anterior segment parameters were measured before and after application of tropicamide (determined by an absence of pupillary light reflex) with the Pentacam system. The Oculus Pentacam is a noninvasive evaluative test of ocular anterior segment topography using Scheimpflug photography. The mechanics of the system have been described in detail elsewhere [16]. Participants were seated comfortably and asked to keep their chin on the chinrest and forehead resting against the forehead strap. Every participant was instructed to keep both eyes open and to fixate on the black target in the center of the blue fixation beam when the instrument was in scan mode. The real-time image on the computer screen was adjusted manually by moving a joystick in the directions indicated on the screen. When the image met *x*-, *y*-, and *z*-plane alignment criteria, the scan was automatically commenced in an automatic release mode. Only scans registering as acceptable (‘‘OK”) on the instrument's “QS” (quality specification) output were used for analysis.

### 2.4. Definitions and Statistical Analysis

Spherical equivalence (SE) was calculated as the spherical power plus half-negative cylinder power. Emmetropia was defined as an SE between −0.5 D and +0.5 D after cycloplegia, myopia as an SE ≤ −0.5 D, and hyperopia as an SE ≥ +0.5 D. The results were expressed as mean ± standard deviation. All statistical evaluations were performed using SAS version 9.2 (SAS Institute). Shapiro-Wilk test was used to assess normal distribution. Levene's test was used to test the variance homogeneity. Paired *t*-test, independent sample *t*-test, and one-way analysis of variance (ANOVA) with pairwise Bonferroni posttests were used to analyze the differences between variables. Wilcoxon signed ranks test and Kruskal-Wallis test were applied when the normality assumption and homogeneity of the variance assumption were violated. Pearson correlation coefficient was used to determine the association between refractive error and biometric parameters. *P* < 0.05 was considered statistically significant. All tests were two-tailed.

## 3. Results

Of the 309 participants examined, 281 children (90.94% of participants) aged 6–13 years (mean, 8.87 ± 1.54 years) were selected for analysis in this study, including 138 girls (49.11%) and 143 boys (50.89%) (Table 1). Twenty-eight children (9.96%) were excluded due to poor cooperation or low scan quality. No subject had a pupil response to light after the application of 5 drops of 0.5% tropicamide. All measurements of the anterior segment were normally distributed both before and after cycloplegia. The overall distributions of measurements before and after cycloplegia, as well changes between the two sessions, are detailed in Table 2.

### 3.1. Relationship between Crystalline Lens and Refractive Status

**Figure 1:**
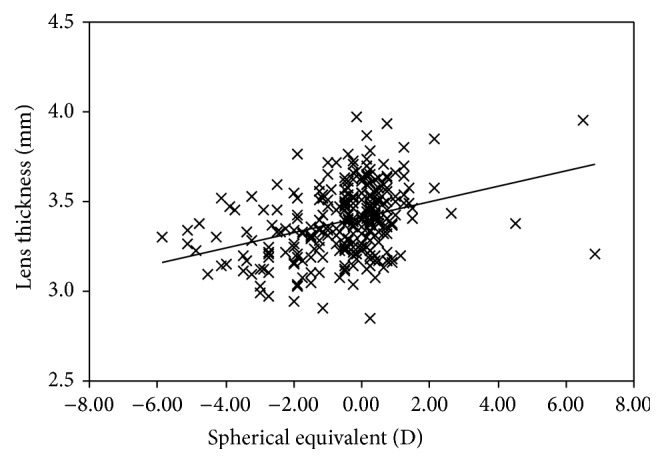
Association between lens thickness and refractive status in the cycloplegia.

**Figure 2:**
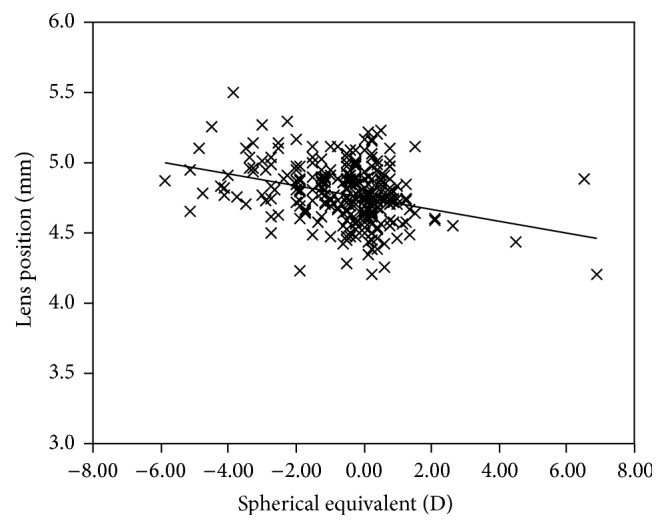
Association between lens position and refractive status in the cycloplegia.

The mean LT and LP in the cycloplegic state were significantly different among the myopic, emmetropic, and hyperopic groups (*P* < 0.01). A positive association was found between the LT and refractive status (age adjusted; *r* = 0.26, *P* < 0.01; Figure 1), while a negative association was noted between the LP and refractive error (age adjusted; *r* = −0.31, *P* < 0.01; Figure 2). The LT significantly decreased with increasing age, from age 6 to 13 years (SE adjusted; *r* = −0.31, *P* < 0.01). No significant difference was found in LT and LP between boys and girls. And there was no significant difference in sex among the myopic, emmetropic, and hyperopic groups. We analyzed the difference in age among the three groups and found the age in the myopic group was different from the emmetropic and hyperopic groups.

### 3.2. Tropicamide-Induced Changes in Anterior Segment Parameters and Their Association with Refractive Status

After application of tropicamide, the values of ACD and ACV increased (both *P* < 0.01), while ACA decreased significantly (*P* < 0.05). The front and back corneal radius and corneal astigmatism were not significantly different from before cycloplegia (*P* > 0.05, resp.). A high correlation was found between ACD and ACV both before and after cycloplegia (*r* = 0.81, *P* < 0.01, and *r* = 0.80, *P* < 0.01, resp.).

Changes in the ACD (ΔACD) and volume (ΔACV) after cycloplegia were less pronounced in the myopes than those in the emmetropes or hyperopes (*P* < 0.05; Table 2). The change of ACA (ΔACA) was different among refractive groups (*P* < 0.05; Table 2). ΔACA of the myopic and emmetropic group had a decrement (*P* = 0.14, *P* < 0.05, resp.), while that of the hyperopic group had an increment (*P* = 0.32). There was no significant difference between genders for ΔACD, ΔACV, and ΔACA in any group (Table 3). No significant difference based on age was found for corneal radius, corneal astigmatism, ACD, ACV, and ACA.

### 3.3. Relationship between Changes in SE (ΔSE) and Changes in Anterior Segment

**Figure 3:**
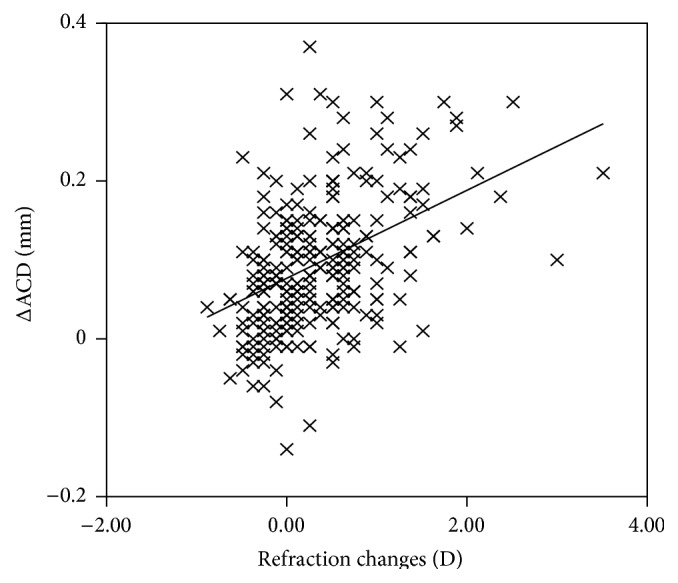
Association between the change of anterior chamber depth and the change of refraction.

Cycloplegia with application of 0.5% tropicamide significantly changed the mean SE from −0.87 D to −0.54 D (*P* < 0.01; Table 1), which was statistically smaller in the myopic group (from −1.93 D to −1.85 D, ΔSE 0.08 ± 0.48 D) than in the emmetropic (from −0.30 D to 0.03 D, ΔSE 0.33 ± 0.53 D) and hyperopic (from −0.30 D to 1.16 D, ΔSE 0.86 ± 0.77 D) groups. However, changes of ACD per diopter in any group were not statistically significant, with myopes presenting 0.16 ± 0.33 mm/D, emmetropes 0.21 ± 0.45 mm/D, and hyperopes 0.20 ± 0.62 mm/D. The association between ΔACD, ΔACV, and ΔSE was demonstrated by Pearson correlation. It was found that ΔSE was positively associated with ΔACD (*r* = 0.41, *P* < 0.01; Figure 3). The weaker association was found between ΔSE and ΔACV (*r* = 0.22, *P* < 0.01).

## 4. Discussion

In the current study, we evaluated the change of anterior segment and refraction and their associations due to cycloplegia in different refractive status. The finding indicated that ACD, ACV, and ACA changed significantly after cycloplegia. And ΔACD was correlated with refraction changes. Myopic children had smaller ΔACD, ΔACV, and ΔACA when compared to emmetropic and hyperopic children.

We found that lenses in the myopic group were significantly thinner than that in the emmetropic and hyperopic group, with LT 3.31 mm in myopes, 3.43 mm in emmetropes, and 3.46 mm in hyperopes. In human eyes, every diopter of reduced accommodation produces only a 0.06 mm decrease in lens thickness [17]. The 0.12 mm decreased in the LT in the myopia would result in an excess −2 D. This was consistent with the average refractive error −1.85 ± 1.25 D in the myopic group. We further took advantage of the Emsley simplified eye model and relevant geometrical optics mechanism to probe the changes of LT as well as LP and their effect on the development of myopia. For eyes with thinner lenses, the focal length would increase due to the reduction lens refractive power. Additionally, the nodal point would have backward movement as LP changed. Both of them could lead to the image of objects located posteriorly to the retina. This can result in hyperopic defocus of retina and then increase the degree of myopia.

Animal experiments have proved that a concave lens could lead to myopia onset and development [18], which was consistent with the theory of hyperopic defocus. Lambert has found that removing the crystalline lens and implanting an intraocular lens in a neonatal monkey eye retarded its axial elongation [19]. This phenomenon may be caused by the relative static intraocular lens which cannot become thinner with age. Gradually, these eyes with such intraocular lenses produced myopic defocus. A longitudinal study in Chinese children revealed that newly developed myopia had slightly lower lens power than in children with persistent emmetropia, when they were still at emmetropic state [20]. Consequently, the crystalline lens could be an important contributor to myopia onset and development and may be a clue to the physician to observe refractive development. However, the children in our study, aged 6 to 13 years, had mild to moderate myopia. Further studies need to be conducted to investigate the role of the crystalline lens in myopia progression, including different degrees of myopia.

We found a significant increase in ACD as well as ACV after application of tropicamide in any group, which was consistent with the result of previous reports [21–23]. However, there was no significant difference in the front and back corneal radii before and after cycloplegia. In sum, the changes in ACD and ACV may be due to the decrease of LT which was caused by axial flattening and backward movement of the lens. Furthermore, a smaller increment in ACD was seen in the myopic group and a greater increment in the emmetropic and hyperopic groups in our study. It may be one of the optical structure basements to insufficient accommodation in myopia. When accommodation occurs, the refractive index of ocular increased due to the increase of LT and decrease of anterior segment. In myopia, it had a smaller change in anterior segment, which may lead to a less increased refractive power during accommodation.

In addition, after relaxing accommodation by tropicamide, the change of refraction was smaller in myopia compared to emmetropia and hyperopia. It was thought that the refraction change after cycloplegia was mainly associated with the decrease of lens power which was due to the decrease of the lens surfaces curvature, lens position, and lens thickness [24]. However, the accompanying changes in anterior chamber depth may also play a role in the refraction change after cycloplegia. In this study, we found a significant change of anterior segment after cycloplegia and found a positive correlation between ΔACD and ΔSE. The probability was significant, although the correlation coefficient was low. ΔACD caused by per diopter change in myopia is slightly smaller. It may prove that the insufficient accommodation in myopia is related to the anterior structure. This may be caused by the thickened ciliary muscle in myopia which had poor contractility and dilatability [25]. As the subjects had mostly mild to moderate myopia in our study, the function of ciliary muscle may not change significantly. A 0.19 mm ΔACD per diopter accommodation was found in our study. This was different from the result of previous studies, which measured the ocular dimensions at different accommodation stimuli [26]. It may be due to the fact that the actual accommodative responses were less than the stimuli especially in myopia.

We also found that ACA changed after cycloplegia and ΔACA differed in different refractive groups. ΔACA in the hyperopic group was a slight increment, whereas in the emmetropic and myopic groups there was a decrement (although the change in the myopic group was statistically insignificant). Contrary to our results, Tsai and associates [27] recently found that the ACA in myopes and emmetropes increased significantly after diagnostic cycloplegia using 1% tropicamide, as observed with the Pentacam system. The discrepancy between their results and ours can be explained by the following. Firstly, the concentrations of tropicamide were different (0.5% in our study and 1% in Tsai et al.'s study), which could cause cycloplegia and pupil dilation to different degrees and result in different changes in anterior lens displacement. Secondly, the baseline anterior segment parameters were different. Subjects in the Tsai et al. study had larger ACA before cycloplegia than ours. Therefore, the value of ΔACA may depend on both the anterior segment configurations at baseline and the response to tropicamide. However, the exact mechanism needs further investigation. Tsai et al. [27] also proved that there was no association between the change of intraocular pressure (IOP) and the change of ACA in school-aged children. So the importance of the change of ACA after cycloplegia in youths is different from that in elders. The relationships between ACA and refractive status and IOP are still not clear.

There were some limitations in this study. First, the pharmacological cycloplegia after application of 0.5% tropicamide may be incomplete. It was thought that good cycloplegia and dilation were achieved with atropine. But it took too long to reach maximum dilation and had more adverse effect [28]. In China, 0.5% tropicamide has been routinely used for refraction or dilated fundus examination in children. For this, the result of our study may be more helpful in clinical practice. Second, commercially available anterior segment measurement instruments could not image the posterior surface of the lens. Further study will be needed to investigate more ocular biometric parameters among more subjects.

In conclusion, children with myopia presented thinner and more posterior lenses, as well as smaller changes of anterior segment parameters and refraction after cycloplegia. The change of ACD was correlated with the change of refraction. This would help in better understanding the relationship between anterior segment and myopia.

## Figures and Tables

**Table 1:** Patient demographics and refractive status.

	Emmetropia	Hyperopia	Myopia
Pre-SE (D)	−0.30 ± 0.56	0.33 ± 1.05	−1.93 ± 1.21

Post-SE (D)	0.03 ± 0.25	1.16 ± 1.23	−1.85 ± 1.25
*n*	104 (37.01%)	58 (20.64%)	119 (42.35%)
Male/female	56/48	29/29	58/61
Age (y)	8.64 ± 1.53	8.12 ± 1.59	9.43 ± 1.32

SE: spherical equivalence.

**Table 2:** Anterior segment parameters before and after cycloplegia among refractive groups.

	Emmetropia	Hyperopia	Myopia

Pre-ACD (mm)	2.93 ± 0.23	2.81 ± 0.23^a^	3.12 ± 0.24^a,b^
Post-ACD (mm)	3.03 ± 0.22	2.99 ± 0.21	3.17 ± 0.23^a,b^
ΔACD	0.10 ± 0.08	0.18 ± 0.10^a^	0.06 ± 0.06^a,b^
*P* value	<0.01	<0.01	<0.01

Pre-ACV (mm^3^)	162.33 ± 22.82	153.90 ± 23.50^a^	178.86 ± 28.63^a,b^
Post-ACV (mm^3^)	174.38 ± 23.28	172.02 ± 23.60	185.10 ± 26.30^a,b^
ΔACV	12.05 ± 10.90	18.12 ± 14.97^a^	6.24 ± 13.23^a,b^
*P* value	<0.01	<0.01	<0.01

Pre-ACA (°)	36.94 ± 4.57	35.02 ± 4.86^a^	38.64 ± 5.15^a,b^
Post-ACA (°)	35.22 ± 7.86	35.87 ± 7.92	37.75 ± 7.66
ΔACA	−1.72 ± 7.38	0.85 ± 6.46^a^	−0.89 ± 6.51
*P* value	<0.05	0.321	0.138
Post-LT (mm)	3.43 ± 0.19	3.46 ± 0.19	3.31 ± 0.18^a,b^
Post-LP (mm)	4.75 ± 0.21	4.72 ± 0.20	4.83 ± 0.20^a,b^

ACD: anterior chamber depth, ACV: anterior chamber volume, ACA: anterior chamber angle, LT: lens thickness, LP: lens position, and Δ: difference between the pre- and postparameter.

^a^ *P* < 0.05 compared with the emmetropia group.

^b^ *P* < 0.05 compared with the hyperopia group.

**Table 3:** Difference in anterior segment parameters between genders among the three groups.

	ΔACD			ΔACV			ΔACA		
	Male	Female	*P*	Male	Female	*P*	Male	Female	*P*
Total	0.097 ± 0.008	0.097 ± 0.007	0.975	10.706 ± 14.336	10.986 ± 12.727	0.863	−0.996 ± 0.595	−0.676 ± 0.564	0.697
Emmetropia	0.101 ± 0.011	0.096 ± 0.011	0.718	11.232 ± 11.400	13.000 ± 10.910	0.412	−2.773 ± 1.003	−0.496 ± 1.025	0.117
Hyperopia	0.188 ± 0.017	0.169 ± 0.020	0.470	19.690 ± 16.935	16.552 ± 12.808	0.430	2.910 ± 1.316	−3.146 ± 0.945	<0.05
Myopia	0.047 ± 0.007	0.063 ± 0.008	0.127	5.707 ± 13.377	6.754 ± 13.179	0.668	−1.233 ± 0.791	−0.564 ± 0.893	0.577

ACD: anterior chamber depth, ACV: anterior chamber volume, ACA: anterior chamber angle, and Δ: difference between pre- and postparameter.
